# DNA Vaccine–Induced Long-Lasting Cytotoxic T Cells Targeting Conserved Elements of Human Immunodeficiency Virus Gag Are Boosted Upon DNA or Recombinant Modified Vaccinia Ankara Vaccination

**DOI:** 10.1089/hum.2018.065

**Published:** 2018-09-20

**Authors:** Xintao Hu, Antonio Valentin, Yanhui Cai, Frances Dayton, Margherita Rosati, Eric G. Ramírez-Salazar, Viraj Kulkarni, Kate E. Broderick, Niranjan Y. Sardesai, Linda S. Wyatt, Patricia L. Earl, Bernard Moss, James I. Mullins, George N. Pavlakis, Barbara K. Felber

**Affiliations:** ^1^ Human Retrovirus Pathogenesis Section, National Cancer Institute, Frederick, Maryland.; ^2^ Human Retrovirus Section, National Cancer Institute, Frederick, Maryland.; ^3^ Inovio Pharmaceuticals, Inc., Plymouth Meeting, Pennsylvania.; ^4^ Laboratory of Viral Diseases, NIAID, Bethesda, Maryland.; ^5^ University of Washington, Seattle, Washington.

**Keywords:** vaccine, immunization, durability, long-lasting, macaque, electroporation, vaccination

## Abstract

DNA-based vaccines able to induce efficient cytotoxic T-cell responses targeting conserved elements (CE) of human immunodeficiency virus type 1 (HIV-1) Gag have been developed. These CE were selected by stringent conservation, the ability to induce T-cell responses with broad human leukocyte antigen coverage, and the association between recognition of CE epitopes and viral control in HIV-infected individuals. Based on homology to HIV, a simian immunodeficiency virus p27^*gag*^ CE DNA vaccine has also been developed. This study reports on the durability of the CE-specific T-cell responses induced by HIV and simian immunodeficiency virus CE DNA-based prime/boost vaccine regimens in rhesus macaques, and shows that the initially primed CE-specific T-cell responses were efficiently boosted by a single CE DNA vaccination after the long rest period (up to 2 years). In another cohort of animals, the study shows that a single inoculation with non-replicating recombinant Modified Vaccinia Ankara (rMVA62B) also potently boosted CE-specific responses after around 1.5 years of rest. Both CE DNA and rMVA62B booster vaccinations increased the magnitude and cytotoxicity of the CE-specific responses while maintaining the breadth of CE recognition. Env produced by rMVA62B did not negatively interfere with the recall of the Gag CE responses. rMVA62B could be beneficial to further boosting the immune response to Gag in humans. Vaccine regimens that employ CE DNA as a priming immunogen hold promise for application in HIV prevention and therapy.

## Introduction

Viral diversity and immune dominance are two major obstacles for the development of an effective human immunodeficiency virus (HIV) vaccine. To address these problems, different approaches have been implemented in the field of vaccine development, including the use of consensus and conserved sequences, center-of-tree (COT) sequences, mosaic immunogens, use of ancestral sequences, or generation of chimeric molecules combining known epitopes.^1–22^ The authors' approach has been to generate immunogens encoding highly conserved sequences within HIV-1 p24^Gag^, and the vaccine comprised seven regions covering 54% of the HIV-1 p24^Gag^ protein.^12–17,23^ The focus was on Gag because it has been shown that T-cell responses targeting epitopes within Gag are beneficial to control viral replication in HIV-1-infected individuals.^12,13,24–29^ Furthermore, the simian immunodeficiency virus (SIV)/macaque model showed that Gag-specific T-cell responses inversely correlate with both peak viral load and chronic viremia, suggesting a protective mechanism of these responses in controlling viremia.^30–34^

A HIV Gag conserved element (CE) DNA vaccine was generated selecting regions characterized by stringent conservation, association with control of viremia in HIV-infected patients, and broad human leukocyte antigen coverage independent of alleles associated with virus control.^12–17^ It was previously shown that macaques immunized with either DNA expressing full-length HIV-1 p55^Gag^ or SIV p57^Gag^ develop primarily T-cell responses targeting variable epitopes within the protein, but only half of the immunized animals developed responses targeting few of the conserved epitopes.^17,23,35^ This finding could be explained by a more promiscuous recognition of the variable epitopes by the macaque major histocompatibility complex (MHC) alleles, together with a restricted distribution of the alleles with high affinity for the peptides encoding the conserved epitopes. Alternatively, the variable epitopes within Gag could interfere with the recognition of the conserved epitopes through a poorly understood mechanism of immune dominance. It was shown that vaccination of both mice and macaques with p24CE DNA induced strong cellular responses targeting the conserved epitopes in all the vaccinated animals,^16,17^ excluding the possibility that the absence of conserved epitopes recognition in macaques immunized with the full-length molecules is due to the lack of the appropriate MHC alleles. Furthermore, it was found that boosting with full-length p55^*gag*^ DNA could efficiently increase the CE-specific responses elicited by CE DNA priming vaccination in all the animals, clearly demonstrating that immune dominance skewed epitope recognition to variable regions when both variable and conserved epitopes are present during the priming of adaptive T-cell responses.^17^ By analogy to HIV, a SIV p27^*gag*^ CE DNA vaccine was developed,^35^ and it was demonstrated that the SIV p27CE DNA vaccine is immunogenic and elicited responses targeting the CE in all macaques. In addition, it was found that a regimen that combines a co-immunization booster vaccination comprising CE and *gag* DNA is the most efficient regimen to elicit CE responses of high magnitude, cytotoxicity, and breadth for both HIV and SIV.^35^ Induction of durable immune responses is a critical feature of a successful vaccine.

The present study shows that the T-cell responses induced by the DNA-based CE vaccine regimens targeting CE within Gag (both HIV and SIV) are long-lasting (around 2 years) and that they are effectively recalled upon a single booster vaccination using CE DNA, indicating the DNA vaccine regimen induced durable memory responses in macaques.

The concept of DNA prime-recombinant Modified Vaccinia Ankara (rMVA) boost (reviewed in Iyer and Amara,^36^ Chea and Amara,^37^ and Garcia-Arriaza and Esteban^38^) has been shown to elicit robust, durable, and protective responses in the SIV/macaque model.^18,39–47^ Importantly, this vaccine regimen also induced promising immune responses in several HIV clinical trials.^48–58^ The CE DNA vaccine is being translated into a clinical trial. There is interest in further optimizing vaccine-induced responses, and thus inclusion of a non-replicating rMVA vector rMVA62B (also called MVA/HIV62B),^59,60^ currently in clinical use in other HIV vaccine studies,^48–51^ could be beneficial as a booster vaccine. Since MVA62B expresses HIV Gag/pol+Env, the possibility that the presence of Env could negatively impact the development of Gag T-cell responses, as has been reported previously,^61–63^ was an important concern. In this context, the vulnerability of the subdominant CE T-cell responses^17^ upon booster vaccinations was examined.

## Materials and Methods

### Ethics statement

All animals were cared for and procedures performed under a protocol approved by the Institutional Animal Care and Use Committee of BIOQUAL, Inc. (animal welfare assurance number A3086-01; protocol number 15-008; and United States Department of Agriculture [USDA] certificate number 51-R0036). The macaques in this study (NCI protocols F26, F30, and F32) were managed according to the animal husbandry program, which aims to provide consistent and excellent care to nonhuman primates in the vivarium. This program operates based on the laws, regulations, and guidelines promulgated by the USDA (*e.g.*, the Animal Welfare Act and its regulations, and the Animal Care Policy Manual), Institute for Laboratory Animal Research (*e.g.*, Guide for the Care and Use of Laboratory Animals, 8th edition), Public Health Service, National Research Council, Centers for Disease Control and Prevention, and the Association for Assessment and Accreditation of Laboratory Animal Care International. The nutritional plan utilized by BIOQUAL, Inc., consisted of twice daily feeding with a Labdiet 5045 High Protein Primate Diet, and food intake was closely monitored by animal research technicians. This diet was also supplemented with a variety of fruits and vegetables as part of the environmental enrichment program established by the veterinary staff and enrichment technician. Pairing of animals as part of the environmental enrichment program was managed by the enrichment technician. All primary enclosures and animal rooms were cleaned daily with water and sanitized at least once every 2 weeks. The macaques (*N* = 20) comprised 19 males and one female (R108, SIV vaccine group) and have been described elsewhere.^17,35^ All vaccinations were performed under anesthesia (ketamine, 10 mg/kg).

### DNA and rMVA vectors

The following plasmids were used in these studies: HIV-1 Gag CE single expression vectors, p24CE1 (plasmid 234H) and p24CE2 (plasmid 235H), encode the two versions of the seven CE identified within p24^Gag16,23^; dual promoter vector p24CE1 + p24CE2 (plasmid 306H) encodes both p24CE immunogens^35^; COT-M p55^*gag*^ (plasmid 222H) and HXB2 p55^*gag*^ (plasmid 114H) DNA encode a full-length HIV-1 Gag representing a center-of-tree sequence and clade B strain,^16,23^ respectively; SIV Gag CE expression vectors, p27CE1 (plasmid 262S) and p27CE2 (plasmid 263S), encode the two SIV CE immunogens^35^; the SIV p57^*gag*^ (plasmid 206S)^64^; and macaque IL-12 DNA (plasmid AG157) serves as a vaccine adjuvant.^65–74^ Endotoxin-free DNAs were prepared according to the manufacturer's protocol (Qiagen, Valencia, CA). Non-replicating rMVA62B encodes HIV Gag/pol (HXB2) and a truncated Env (ADA) lacking the cytoplasmic tail has been described previously.^59,60^

### Vaccination of rhesus macaques

The animals were primed with HIV-1 or SIV Gag CE DNA followed by booster vaccinations with DNA vectors encoding full-length *gag* or a combination of CE and *gag* as part of previous studies.^23,35^ Table 1 details the vaccination scheme. After around 1.5–2 years of rest, the animals immunized with HIV-1 vectors received a single booster vaccination with either p24CE DNA (*n* = 8) or 10^8^ pfu of rMVA62B (*n* = 6). Animals immunized with SIV CE DNA vaccine regimen were boosted with p27CE DNA. Each DNA vaccine mixture contained 0.2 mg of macaque interleukin-12 (IL-12) DNA and was formulated in water. Plasmid DNA vaccines were delivered via the intramuscular (i.m.) route followed by *in vivo* electroporation (i.m./EP) using the Elgen 1000 device (Inovio Pharmaceuticals, Inc., Plymouth Meeting, PA). rMVA62B vaccine (1 mL) was delivered i.m. with a needle/syringe.

**Table 1.** Vaccination scheme

*Animal ID*	*CE DNA vaccine regimen*	*Rest time (months)*	*Booster vaccine*
L862	HIV CE DNA prime/*gag* DNA boost^a^	24	HIV CE DNA
M166	HIV CE DNA prime/*gag* DNA boost^a^	24	HIV CE DNA
M695	HIV CE DNA prime/*gag* DNA boost^a^	24	HIV CE DNA
R279	HIV CE DNA prime/*gag* DNA boost^a^	24	HIV CE DNA
R315	HIV CE DNA prime/*gag* DNA boost^a^	19	HIV CE DNA
P302	HIV CE DNA prime/*gag* DNA boost^a^	19	HIV CE DNA
P307	HIV CE DNA prime/*gag* DNA boost^a^	19	HIV CE DNA
P308	HIV CE DNA prime/*gag* DNA boost^a^	19	HIV CE DNA
L986	SIV CE DNA prime/*gag* DNA boost^b^	22	SIV CE DNA
R108	SIV CE DNA prime/*gag* DNA boost^b^	22	SIV CE DNA
R677	SIV CE DNA prime/*gag* DNA boost^b^	15	SIV CE DNA
R682	SIV CE DNA prime/*gag* DNA boost^b^	15	SIV CE DNA
R683	SIV CE DNA prime/*gag* DNA boost^b^	15	SIV CE DNA
R684	SIV CE DNA prime/*gag* DNA boost^b^	15	SIV CE DNA
5698	HIV CE DNA prime/CE + *gag* DNA boost^b^	17	rMVA62B
5701	HIV CE DNA prime/CE + *gag* DNA boost^b^	17	rMVA62B
5702	HIV CE DNA prime/CE + *gag* DNA boost^b^	17	rMVA62B
5699	HIV CE DNA prime/CE + *gag* DNA boost^b^	19	rMVA62B
5703	HIV CE DNA prime/CE + *gag* DNA boost^b^	19	rMVA62B
5700	HIV CE DNA prime/CE + *gag* DNA boost^b^	19	rMVA62B

^a^ Described in Kulkarni *et al*.^17^

^b^ Described in Hu *et al*.^35^

CE, conserved element; HIV, human immunodeficiency virus; SIV, simian immunodeficiency virus; rMVA, recombinant Modified Vaccinia Ankara.

### Intracellular cytokine staining

Ficoll-Hypaque (Histopaque; Sigma–Aldrich, St. Louis, MO) isolated peripheral blood mononuclear cells (PBMC) were cultured on 96-well plates in the presence of SIV or HIV peptide pools at a final concentration of 1 μg/mL for each peptide. Peptide pools covering the complete SIV/HIV CE or each of the seven individual CE were prepared including 15-mer peptides overlapping by 11 amino acids (AA) and 10-mer peptides overlapping by nine AA.^17,35^ Putative T-cell responses recognizing neoantigens generated by the linkers between the individual CE were analyzed using individual 15-mer peptides covering each junction and a pool of all the junction peptides. Peptide stimulation was performed for 12 h in the presence of the protein transport inhibitor monensin (GolgiStop; BD Biosciences, San Jose, CA) and anti-CD107a, monoclonal antibody (clone eBioH4A3; eBioscience, San Diego, CA). Antigen-specific T-cell responses were measured by combining surface and intracellular cytokine staining, as detailed elsewhere.^18,20,30,35,75^ The following antibodies were used in the cocktail for surface staining: CD3-APCCy7 (clone SP34-2), CD4-V500 (clone L200), and CD95-FITC (clone DX2; BD Pharmingen, San Diego, CA); CD8-Alexa Fluor-405 (clone 3B5; Invitrogen, Carlsbad, CA); and CD28-PerCP Cy5.5 (clone CD28.2; BioLegend, San Diego, CA). After cell permeabilization, intracellular staining was performed using interferon gamma (IFN-γ)-PE Cy7 (clone B27; BD Pharmingen), granzyme B-APC or PE (clone GB12; Invitrogen), and Perforin-FITC (clone Pf-344; Mabtech AB, Stockholm, Sweden) antibodies. As negative and positive controls, PBMC were cultured in medium without peptide pools or in the presence of a commercial mix of PMA and calcium ionophore (Sigma–Aldrich), respectively. Samples were acquired on a LSR II or Fortessa flow cytometer (BD Biosciences), and the data were analyzed using FlowJo software (Tree Star, Inc., Ashland, OR). Samples were considered positive if the frequency of cytokine-positive T cells was twofold higher than that of the unstimulated medium-only control and >0.01 after subtracting the medium control value.

### Epitope mapping by macaque IFN-γ enzyme-linked immunospot assay

Epitope mapping of vaccine-induced responses was performed with cryopreserved PBMC using a macaque IFN-γ enzyme-linked immunospot (ELISpot) assay (cat. no. 3421M-4AST-4; Mabtech AB) with peptides spanning HIV p24^Gag^ following the manufacturer's instructions. Lymphocytes were thawed in RPMI-1640 medium supplemented with 10% fetal calf serum and penicillin/streptomycin (R-10 medium), and rested in R-10 medium with 10 IU/mL of DNase I (Roche, Indianapolis, IN) at 37°C for 1 h. Cells were seeded onto 96-well plates (2 × 10^5^ cells/well) in duplicate wells and stimulated for 16 h at 37°C with individual peptides covering the HIV-1 HXB2 p24^Gag^ (15-mer peptides overlapping by 11 AA; Infinity Biotech Research & Resource, Inc., Aston, PA) at a final concentration of 1 μg/mL. As positive control, an anti-CD3 antibody (clone CD3-1) provided by the kit was used in duplicate wells. After incubation, the plates were washed to remove the cells. Then, 100 μL of biotin-labeled detection antibody (7-B6-1) was added to each well and incubated for 2 h at room temperature (RT). After an additional wash, 100 μL of streptavidin-ALP was added to each well and incubated for 1 h at RT. The plates were developed with 100 μL of 5-bromo-4-chloro-3-indolyl phosphate/Nitroblue Tetrazolium substrate (BCIP/NBT; Bio-Rad Laboratories, Inc., Irvine, CA). Spots were counted in an automated ELISpot reader system (CTL Analyzers LLC, Cleveland, OH) and analyzed using ImmunoSpot software. The responses are expressed as spot-forming cells per million PBMC.

### Perforin detection by ELISpot

Perforin release by antigen-specific T cells was monitored in cryopreserved PBMC stimulated with a Gag CE peptide pool (mixture of 15-mer and 10-mer; see above) covering the complete CE immunogen by ELISpot (cat. no. 3465-4APW-2; Mabtech AB) according to the manufacturer's instructions.

### Statistical analyses

Statistical analyses were performed with Prism v7.0 (GraphPad Software, Inc., La Jolla, CA).

## Results

### DNA-based prime-boost vaccine regimens elicit long-lasting CE-specific T-cell responses

**Figure 1.**
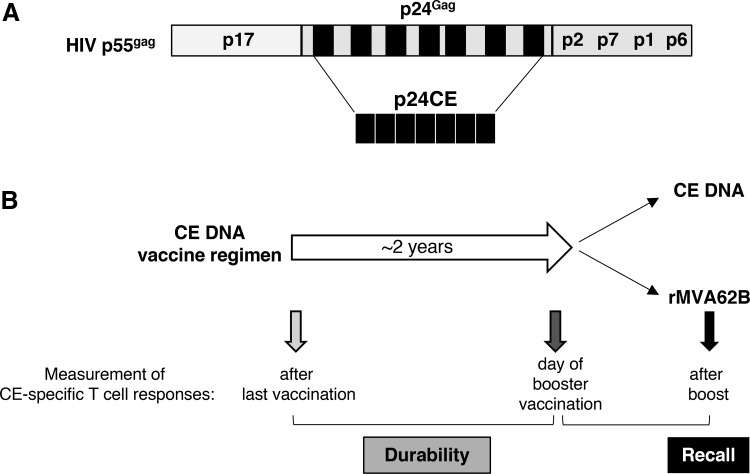
DNA-based vaccine regimens induce long-lasting conserved element (CE)-specific T-cell responses. **(A)** Schematic representation of the seven CE identified within human immunodeficiency virus type 1 (HIV-1) p24^Gag^ protein. The CE immunogen comprises seven CE, separated by amino acid linkers, and are collinearly arranged.^16^ The p24CE vaccine comprises two CE immunogens (p24CE1 and p24CE2) differing by one AA per CE. The simian immunodeficiency virus (SIV) p27CE immunogen was generated by analogy to HIV p24CE.^35^ **(B)** Scheme of the experimental design for this booster immunizations. All macaques were immunized with the CE DNA vaccine comprising a combination of CE DNA as prime and full-length *gag* DNA as boost. The durability of the DNA-induced CE-specific T-cell responses were measured after a rested for a period of around 2 years. Recall responses were measured upon a single CE DNA or recombinant Modified Vaccinia Ankara (rMVA62B) vaccination.

The key immunogens in the vaccine regimens described in this work are DNA plasmids encoding CE within HIV-1 p24^Gag^ and the homologous SIV p27^Gag^, termed HIV p24CE and SIV p27CE, respectively (Fig. 1A), described elsewhere.^16,23,35^ The aim of this study was to determine the durability of the CE-specific T-cell responses elicited by DNA vaccine regimens consisting of sequential vaccinations with CE as prime followed by full-length Gag as boost (Fig. 1B). CE-specific T-cell responses were measured around 1.5–2 years later to determine longevity of the responses and after a single booster vaccination with either CE DNA or rMVA62B to measure recall responses.

It was previously found that breadth and hierarchy of epitope recognition within Gag is determined by the priming vaccination. It was shown that macaques primed with CE DNA develop robust responses to the conserved subdominant CE and that boosting with full-length *gag* DNA or CE + *gag* DNA was critical to increase the magnitude of the CE-specific responses reaching similar levels,^17,35^ while it also induced T-cell responses outside of CE in Gag.^17^ No significant difference was observed in the magnitude or breadth of the CE responses as a function of the number of CE DNA priming or *gag* DNA booster vaccinations. Two CE DNA priming vaccinations followed by a single *gag* DNA booster vaccination elicited maximal responses that could be recalled by additional vaccinations but did not further increase the magnitude.^17,35^ This allowed macaques that had received the CE DNA vaccine regimen to be combined into the cohorts detailed in Table 1. All animals were vaccinated with the HIV or SIV CE DNA vaccine regimen using i.m. electroporation as the DNA vaccine delivery method.^76–79^

**Figure 2.**
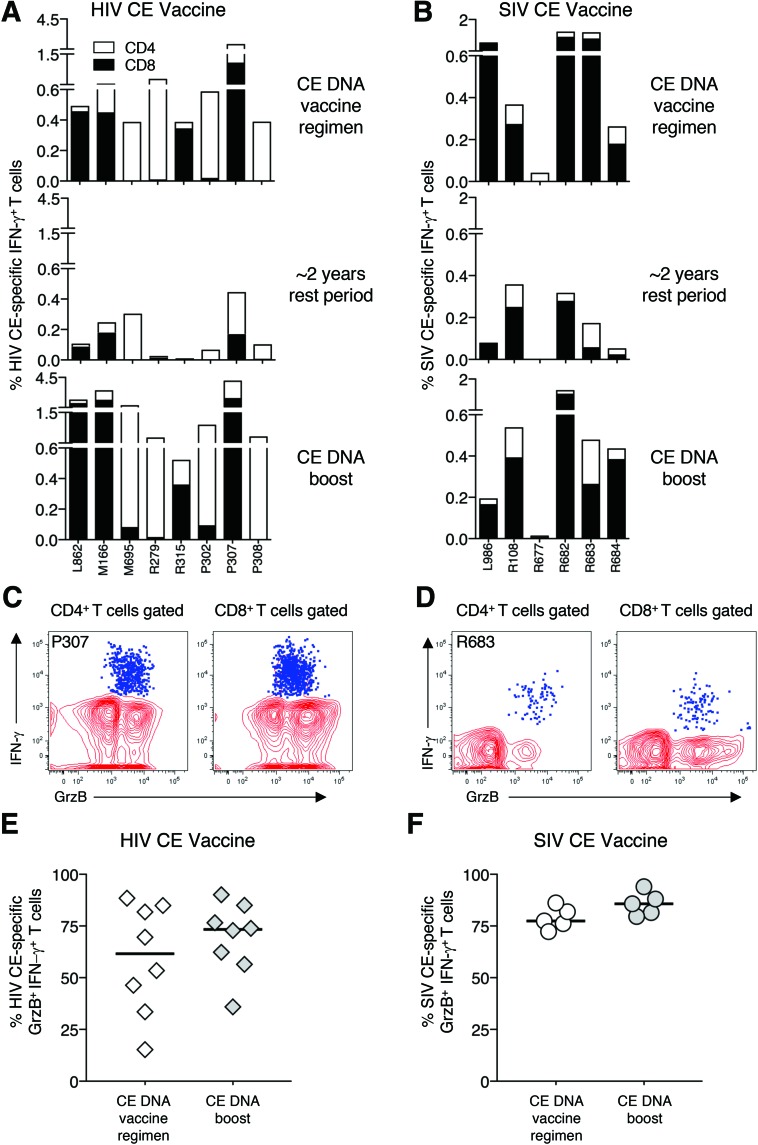
Durability of CE-specific T-cell responses and recall upon a single CE DNA vaccination after around a 2-year rest period. **(A** and **B)** CE-specific CD4^+^ (*open bars*) and CD8^+^ (*black bars*) interferon gamma (IFN-γ)^+^ T-cell responses monitored by flow cytometry are shown from macaques immunized with HIV CE **(A)** or SIV CE **(B)** after a CE DNA vaccination regimen (*upper panels*), after the rest period at the time of boost (*middle panel*), and 2 weeks after the booster immunization (*lower panels*). **(C** and **D)** Dot plots showing the granzyme B (GrzB) content of IFN-γ^+^ CE-specific CD4^+^ and CD8^+^ T cells (*blue dots*) from a representative macaque immunized **(C)** with HIV (macaque P307) and **(D)** SIV CE (macaque R683). **(E** and **F)** The percentage of GrzB^+^ within the CE-specific T cells for the HIV CE **(E)** and SIV CE **(F)** immunized macaques is shown after the CE DNA vaccine regimen and after the single CE DNA boost administered after the long rest period. Due to the very low levels of T-cell immunity, macaque R677 could not be analyzed. Color images available online at www.liebertpub.com/hum

Macaques were immunized with either the HIV-1 CE DNA regimen (Fig. 2A; *n* = 8) or the SIV CE DNA regimen (Fig. 2B; *n* = 6), detailed in Table 1. After the last DNA vaccination, a high magnitude of CE-specific T-cell responses, mediated by both CD4^+^ and CD8^+^ T lymphocytes, was found in all animals (Fig. 2A and B, top panels), except R677 from SIV CE group^35^ that throughout the complete vaccination schedule failed to mount a significant anti-Gag T-cell immune response. Long-lasting immunity is an important component of protective vaccines. Therefore, to monitor the longevity of CE-specific T-cell responses, all macaques were analyzed at around 1.5–2 years after the last DNA vaccination. Persistent CE-specific T-cell responses were found in the blood of animals vaccinated with HIV-1 DNA (Fig. 2A, middle panel) and with SIV CE DNA (Fig. 2B, middle panel).

### CE-specific memory T-cell responses are efficiently boosted by a single CE DNA immunization

After around a 1.5- to 2-year rest period, the ability of a single CE DNA booster vaccination to recall CE-specific T-cell immunity was tested. The HIV CE DNA booster vaccination efficiently expanded the pre-existing CE-specific responses, reaching up to 4.2% CE-specific T cells in the blood of macaque P307, measured 2 weeks later (Fig. 2A, bottom panel; range 0.5–4.2% CE-specific T cells). This increase was significant (paired *t*-test, *p* = 0.008) comparing the levels before and after vaccination, indicating efficient recall of the CE-specific responses. Similarly, the SIV CE DNA booster vaccination significantly increased (paired *t*-test, *p* = 0.031) the magnitude of the responses in all macaques, except R677 (Fig. 2B, bottom panel; range 0.01–1.5% CE-specific T cells).

It was noted that the distribution of CE-specific CD4^+^ and CD8^+^ T cells after the initial CE DNA vaccination and after the CE DNA boost remained similar in both cohorts, with some animals showing mainly CD4^+^ and others mainly CD8^+^ T-cell responses. The study also analyzed by flow cytometry the cytotoxic potential of the T-cell targeting the conserved epitopes before and after the CE DNA booster vaccination. The granzyme B (GrzB) content of the CE-specific CD4^+^ and CD8^+^ T cells from one representative animal from each vaccine group is shown (Fig. 2C and D). The data demonstrate that the T cells targeting the CE epitopes are characterized by a high frequency of cytotoxic cells, for either HIV or SIV, during the complete observation period. The percentage of CE-specific T cells harboring GrzB was consistently >70% after each vaccination in SIV p27CE DNA immunized animals, while animals immunized with the HIV p24CE DNA showed a wider distribution in the GrzB content of the CE-specific T cells, reflecting the dominant CD4^+^ T-cell responses in some of the animals included in this group (Fig. 2E and F). Together, these data demonstrate that the CE DNA vaccine regimen induced potent CE-specific cytotoxic memory T-cell responses.

To understand whether boosting after an extended rest period alters the breadth of the CE responses (number of CE recognized), the CE of all vaccinees was mapped by testing T-cell responses to peptide pools covering individual CE for the HIV CE (Table 2) and the SIV CE (Table 3) cohorts. The same overall breadth of the CE responses was found within an animal. These data showed that the responses against different CE established by the initial vaccine regimen were not appreciably altered by later boosting.

**Table 2.** Comparison of the CE response breadth after HIV CE DNA vaccination administered after a long rest period

		*Responses to individual CE*							
*Animal ID*	*Vaccine*	*CE1*	*CE2*	*CE3*	*CE4*	*CE5*	*CE6*	*CE7*	*No. of positive CE*
L862	CE/*gag* DNA^a^		+	+	+	+			4
	CE DNA boost		+	+	+	+			4
M166	CE/*gag* DNA^a^		+			+	+		3
	CE DNA boost		+			+	+		3
M695	CE/*gag* DNA^a^					+	+		2
	CE DNA boost			+		+	+		3
R279	CE/*gag* DNA^a^			+	+	+	+		4
	CE DNA boost				+		+		2
R315	CE/*gag* DNA^a^					+		+	2
	CE DNA boost					+			1
P302	CE/*gag* DNA^a^					+	+		2
	CE DNA boost					+	+		2
P307	CE/*gag* DNA^a^			+		+			2
	CE DNA boost			+		+			2
P308	CE/*gag* DNA^a^			+					1
	CE DNA boost			+					1

^a^ CE mapping data described in Kulkarni *et al*.^17^

**Table 3.** Comparison of CE response breadth after SIV CE DNA booster vaccination administered after a long rest period

		*Responses to individual CE*							
*Animal ID*	*Vaccine*	*CE1*	*CE2*	*CE3*	*CE4*	*CE5*	*CE6*	*CE7*	*No of positive CE*
L986	CE/*gag* DNA^a^					+	+		2
	CE DNA boost					+	+		2
R108	CE/*gag* DNA^a^					+	+		2
	CE DNA boost					+	+		2
R677	CE/*gag* DNA^a^			+					1
	CE DNA boost			+					1
R682	CE/*gag* DNA^a^			+		+	+		3
	CE DNA boost			+		+	+		3
R683	CE/*gag* DNA^a^			+		+			2
	CE DNA boost			+		+			2
R684	CE/*gag* DNA^a^	+	+	+		+			4
	CE DNA boost	+	+	+		+			4

^a^ CE mapping data described in Hu *et al*.^35^

### A single MVA immunization efficiently boosts the CE-specific T-cell responses

Next, the possibility was explored of combining the HIV p24 CE DNA vaccine with a different kind of booster vaccination, in this case a viral vector, that may target a broader cell population and augment the T-cell responses induced by DNA vaccination regimens. This vaccine regimen was tested using rMVA62B expressing HIV Gag/pol + Env. This allowed the study to test whether the simultaneous production of Env and Gag impairs the ability of the vector to boost efficiently the CE-specific CTL responses. Of note, a strong dominant effect of Env over Gag resulting in dampening the Gag T-cell response development has previously been reported.^61–63^

**Figure 3.**
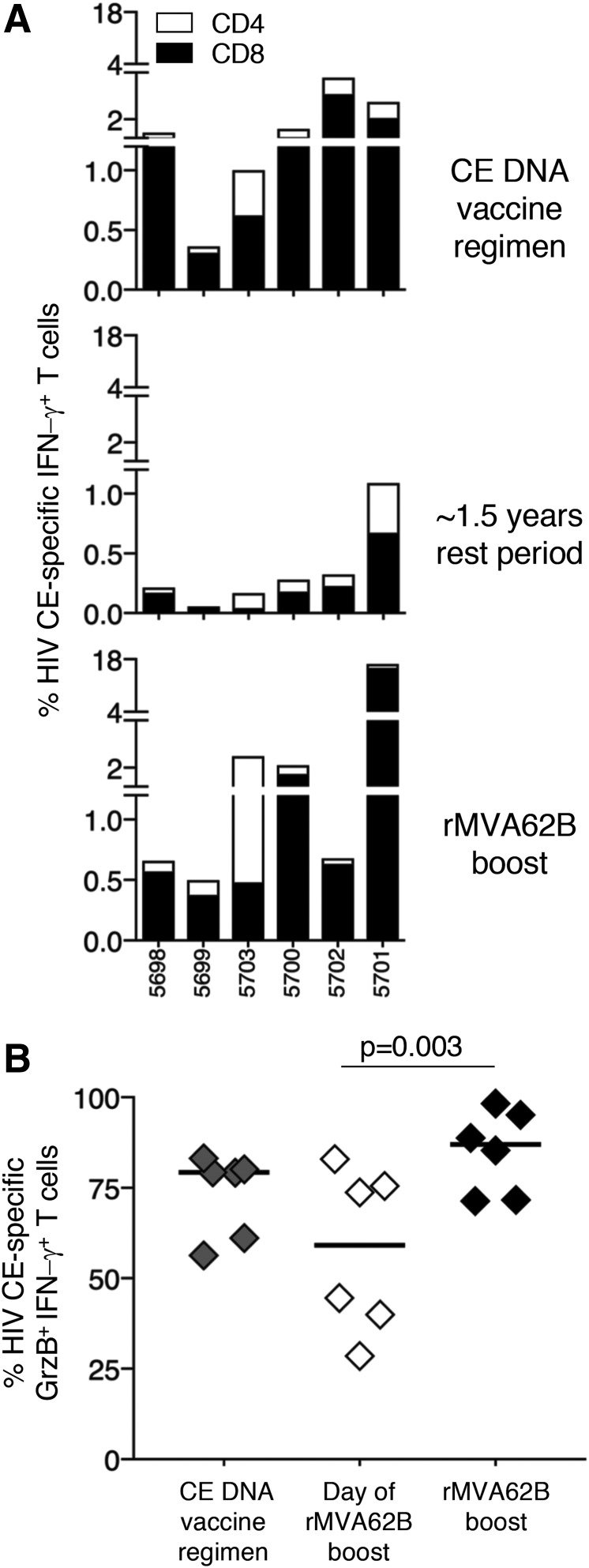
Increase of CE-specific T-cell responses after rMVA62B boost administered after a long rest period. **(A)** Six macaques were immunized with the HIV CE DNA vaccine regimen (Table 1) and, after a 1.5-year rest period, received a single immunization with rMVA62B. **(A)** CE-specific CD4^+^ (*open bars*) and CD8^+^ (*black bars*) IFN-γ^+^ T cells are shown in peripheral blood mononuclear cells collected 2 weeks after the last DNA vaccination (*upper panel*), around 1.5 years later at the time of rMVA vaccination (*middle panel*), and after the rMVA62B boost (*lower panel*). **(B)** Percentage of GrzB^+^ within the CE-specific T cells was measured after CE DNA vaccination, the long rest period, and rMVA62B boost. The *p*-value is from analysis of variance with Dunn's multiple comparisons test.

Six macaques (Table 1) previously vaccinated with the HIV CE DNA regimen that developed robust CE-specific T-cell responses (Fig. 3A, top panel) were enrolled in a booster vaccination protocol using a single inoculation of the rMVA62B administered 17 (three animals) and 19 (three animals) months after the last DNA vaccination. All macaques were still positive for HIV CE-specific T-cell responses at the day of rMVA62B inoculation, with macaque 5701 having >1% of total circulating CD3^+^ T lymphocytes directed to CE (middle panel). Upon a single rMVA62B vaccination, the CE-specific T cells significantly (*p* = 0.031) increased (bottom panel), reaching levels up to 17% of total circulating T cells in macaque 5701. The cytotoxicity of the CE-specific T cells was also evaluated at these three time points by measuring their GrzB content upon peptide stimulation. Potent levels of GrzB^+^ CE-specific T cells were found at peak after CE DNA vaccination (median 79% of CE-specific T cells) that persisted upon the long-term rest period (median 59% of CE-specific T cells; Fig. 3B). rMVA booster vaccination resulted in a significant (*p* = 0.003) increase (median 87%) of GrzB^+^ CE-specific T cells (Fig. 3B), reaching similar levels of cytotoxicity compared to the CE DNA vaccination. The increase in the CE-specific T-cell responses in these animals showed that the long-lasting memory responses elicited by the initial DNA vaccination were rapidly recalled upon new encounter with the Gag antigen expressed by rMVA62B. The robust increase in the CE-specific responses in all animals further indicated that the presence of Env in the rMVA62B vector did not have a negative impact on the recall of the CE-specific T cells.

It has previously been shown that the breadth of vaccine-induced responses was increased in the initial CE + *gag* DNA booster immunizations.^35^ Next, it was determined whether the increase in CE responses upon rMVA62B booster vaccination further broadened the number of CE recognized in each immunized animal. It was found that the CE responses established by the initial CE DNA vaccine regimen were not significantly broadened by boosting with rMVA62B. A summary of these data is presented in Table 4.

**Table 4.** Comparison of the HIV CE response breadth after rMVA62B booster vaccination

		*Responses to individual CE*							
*Animal ID*	*Vaccine*	*CE1*	*CE2*	*CE3*	*CE4*	*CE5*	*CE6*	*CE7*	*No of positive CE*
5698	CE/CE + *gag* DNA^a^		+	+		+			3
	rMVA boost		+	+		+			3
5699	CE/CE + *gag* DNA^a^		+			+			2
	rMVA62B boost		+			+			2
5703	CE/CE + *gag* DNA^a^			+		+	+		3
	rMVA62B boost		+	+		+	+		4
5700	CE/CE + *gag* DNA^a^			+		+	+		3
	rMVA62B boost			+		+	+		3
5701	CE/CE + *gag* DNA^a^		+			+	+		3
	rMVA62B boost		+		+	+	+		4
5702	CE/CE + *gag* DNA^a^		+			+			2
	rMVA62B boost		+			+			2

^a^ CE mapping data described in Hu *et al*.^35^

### CE-specific T-cell responses are characterized by a cytotoxic phenotype

**Figure 4.**
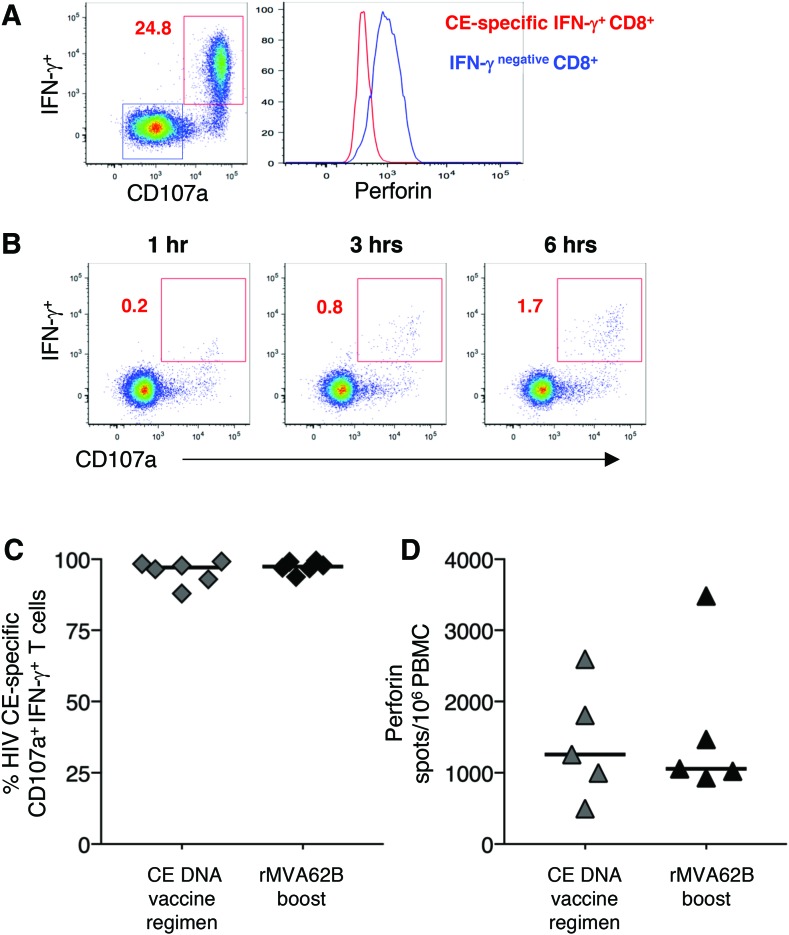
Cytotoxic phenotype of the CE-specific T-cell responses. **(A)** Dot plot showing the frequency of CE-specific CD8^+^ T cells after the rMVA62B boost and a histogram overlay showing the perforin content of the CE-specific IFN-γ^+^ (*red line*) and the general IFN-γ^–^ (*blue line*) in general CD8^+^ T cells are shown from a representative animal (macaque 5701). **(B)** Dot plots showing the kinetics of IFN-γ production and degranulation (CD107a) of the CE-specific T-cell responses at the indicated time points (macaque 5703). **(C)** Percentage of CD107a^+^ degranulating cells from the CE-specific T lymphocytes after DNA vaccination and rMVA62B boost. **(D)** Frequency of perforin secreting CE-specific T cells measured by enzyme-linked immunospot assay shown after DNA vaccination and rMVA62B booster vaccination. No cells were available from macaque 5702. Color images available online at www.liebertpub.com/hum

The study also analyzed the cytotoxic potential of the CE responses induced by rMVA booster vaccination by monitoring their GrzB and perforin content, as well as the ability to degranulate after stimulation with CE peptide pools (Fig. 4A). CE-specific T cells were highly positive for CD107a expression after peptide stimulation, shown for a representative animal (macaque 5701), indicative of their ability to release cytotoxic granules (Fig. 4A, left plot). This response resulted in the release of perforin from the cells, making the detection of perforin in peptide-stimulated T cells not a reliable measurement by flow cytometry. In fact, the perforin content of CE-specific CD107a^+^ CD8^+^ T cells was lower than in the CD107a^–^ general CD8^+^ population within the same sample (Fig. 4A, right plot), suggesting that perforin is lost after active degranulation triggered by specific engagement of the T-cell receptor. Analysis of the kinetics of degranulation (CD107a surface expression) after stimulation with CE-specific peptide pools (Fig. 4B) demonstrated that active degranulation was already present 1 h after stimulation. The study analyzed by flow cytometry the CD107a expression in the IFN-γ^+^ CE-specific T cells 2 weeks after the last DNA vaccination and after the boost with rMVA62B (Fig. 4C), and found that both vaccinations elicit T-cell responses that actively degranulate after T-cell receptor stimulation. Therefore, to monitor perforin release accurately, perforin was analyze in the extracellular compartment using an ELISpot assay. Perforin-secreting cells were measured for all the animals after the CE DNA vaccine regimen and upon a single rMVA62B boost (Fig. 4D). The macaques showed similar levels of perforin-secreting T cells after stimulation with peptides covering the CE epitopes at both time points monitored, indicating that the CE DNA priming vaccination regimen and MVA62B boosting were equally potent in inducing cytotoxic CE-specific T cells.

### Mapping of the HIV CE epitopes

Epitope mapping of HIV CE responses was performed by ELISpot with 55 individual peptides (15-mer overlapping by 11 AA) covering HIV p24^Gag^. Mapping was performed in six animals after the CE DNA vaccination and after the rMVA62B boost (Table 5). This analysis identified immunogenic peptides within five CE: CE2 (peptides 41 and 43/44), CE3 (peptides 49, 50/51), CE4 (peptides 58, 59/60), CE5 (peptides 65/66 and 67–69), and CE6 (peptides 75/76). Thus, CE2, CE4, and CE5 appeared to have two epitopes, whereas CE3 and CE6 had one epitope. Importantly, this analysis also showed that the rMVA62B boost induced responses to peptides 53/54, which lie in the variable region between CE3 and CE4 in four of the six animals. Responses to this variable region were also found in macaque 5701 after the CE DNA vaccination and boosting with rMVA62B, which expanded responses to this region by 8- to 11-fold. Thus, rMVA62B expressing Gag protein is able to induce responses to variable regions outside of the CE in four animals (5698, 5699, 5703, and 5702).

**Table 5.** Positive HIV peptides after HIV CE DNA vaccination and after rMVA62B boost

			*Gag peptides with positive ELISpot responses*											
			*5698*		*5699*		*5700*		*5701*		*5702*		*5703*	
*Gag Peptide*^a^			*CE +* gag	*MVA*	*CE +* gag	*MVA*	*CE +* gag	*MVA*	*CE +* gag	*MVA*	*CE +* gag	*MVA*	*CE +* gag	*MVA*
41	EKAFSPEVIPMFSAL	CE2	+	+		+			+	+	+	+		
43	IPMFSALSEGATPQD	CE2	+	+					+	+	+	+		
44	SALSEGATPQDLNTM	CE2	+	+					+	+	+	+		
49	GHQAAMQMLKETINE	CE3		+										
50	AMQMLKETINEEAAE	CE3	+	+			+	+				+	+	+
51	LKETINEEAAEWDRV	CE3	+	+										
53	AAEWDRVHPVHAGPI	Variable^b^		+		+			+	+		+		
54	DRVHPVHAGPIAPGQ	Variable^b^		+		+			+	+		+		+
58	REPRGSDIAGTTSTL	CE4			+	+	+	+						
59	GSDIAGTTSTLQEQI	CE4												+
60	AGTTSTLQEQIGWMT	CE4							+	+			+	+
65	PVGEIYKRWIILGLN	CE5									+	+		
66	IYKRWIILGLNKIVR	CE5				+	+	+			+	+		
67	WIILGLNKIVRMYSP	CE5					+	+	+	+				
68	GLNKIVRMYSPTSIL	CE5	+	+			+	+	+	+	+		+	
69	IVRMYSPTSILDIRQ	CE5					+	+			+	+	+	
75	VDRFYKTLRAEQASQ	CE6					+	+					+	
76	YKTLRAEQASQEVKN	CE6					+	+						

^a^ Number of HIV HXB2 p55^Gag^ peptide (15-mer overlapping by 11 AA).

^b^ Peptides covering the variable region between CE3 and CE4.

Together, these data showed that rMVA62B vaccination increased the magnitude and cytotoxicity of the CE-specific T-cell responses (Fig. 4) and did not significantly expand the number of epitopes recognized (Table 4). The ability of *gag* DNA or rMVA62B to induce responses outside of the CE (Table 5), despite CE priming, indicated that while the dominant recognition of CE is not lost by boosting with Gag antigen, it is also important to utilize CE as a priming immunogen, as these regions are normally subdominant when initially presented as part of the full-length antigen.

### Analysis of T-cell responses targeting the linkers between the CE

**Figure 5.**
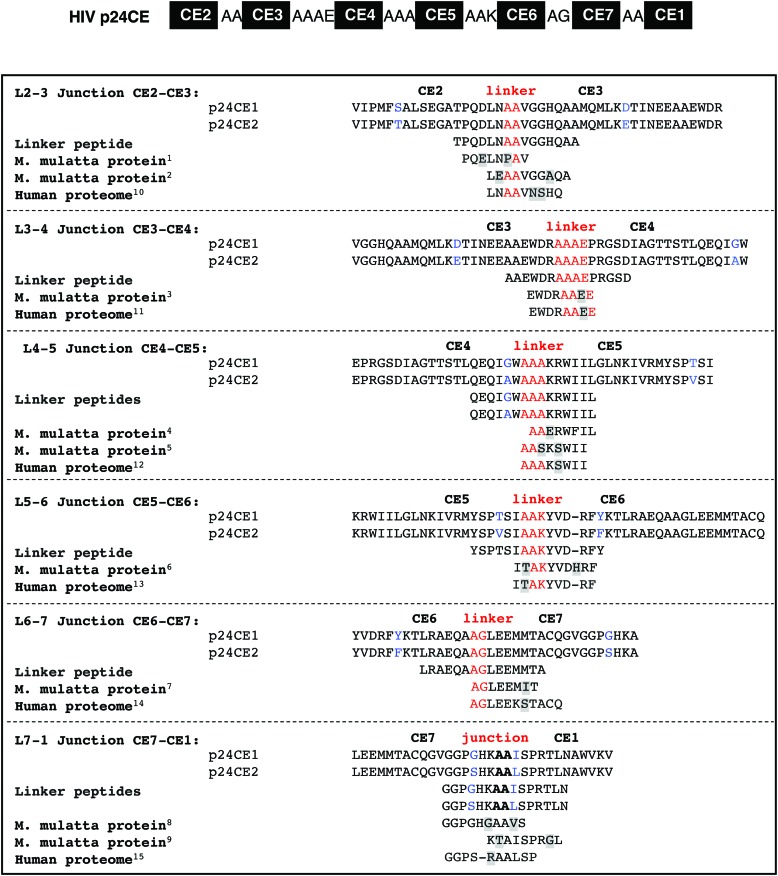
Alignment of the CE junctional regions with predicted macaque and human proteins. The sequence of adjacent CE together with their linkers are shown in the *top panel*, and the sequences are listed in the *bottom panel*. The toggle AA (one per CE) differentiating p24CE1 and p24CE2 are shown in *blue*, while the linker residues are shown in *red*. The sequence of the synthetic peptides covering the linkers was used to analyze T-cell responses potentially targeting neo-antigens outside the CE, and the sequence of predicted *Macaca mulatta* proteins of at least eight AA in length and with greatest homology to these regions are shown at the bottom of each alignment. Mismatched residues are highlighted in *yellow*; a gap indicates missing AA. The identified *M. mulatta* and human proteins are listed: ^1^NP_001248675.1 cyclin-dependent kinase 12; ^2^XP_014986825.1 PREDICTED: xylulose kinase isoform X2; ^3^XP_014995223.1 PREDICTED: E3 ubiquitin-protein ligase MARCH6 isoform X2; ^4^XP_014983460.1 PREDICTED: retrotransposon gag domain-containing protein 1 isoform X1; ^5^XP_014978728.1 PREDICTED: C3 and PZP-like alpha-2-macroglobulin domain-containing protein 8; ^6^XP_015001479.1 PREDICTED: arf-GAP with SH3 domain, ANK repeat and PH domain-containing protein 1 isoform X6; ^7^XP_014973010.1 PREDICTED: interleukin-11 receptor subunit alpha isoform X3; ^8^XP_014979999.1 PREDICTED: choriogonadotropin subunit beta; ^9^XP_014998908.1 PREDICTED: leucine-rich repeat-containing protein 16B isoform X7; ^10^Zinc finger protein 180 isoform 1: NP_037388.3; ^11^E3 ubiquitin-protein ligase isoform X5: XP_011512238.1; ^12^C3 and PZP-like alpha-2-macroglobulin-domain-containing protein 8 isoform x2: XP_011526220.1; ^13^arf-GAP with SH3 domain, ANK repeat and PH domain-containing protein 1 isoform: NP_001234925.1; ^14^importin-5: NP_002262; ^15^junctional protein associated with coronary artery disease isoform X2: XP_011517911.1. Color images available online at www.liebertpub.com/hum

Because the CE DNA vaccine is being developed for a clinical trial, there was also concern over whether peptides spanning the CE were immunogenic. The seven CE identified within HIV p24^Gag^ were connected by AA linkers designed to maximize their proteolytic processing into peptides and binding to MHC molecules for efficient presentation to T cells and avoidance of neo-antigens.^80,81^ There is a possibility that the linkers between the CE generate neo-epitopes that could be recognized by the immune system inducing T-cell responses outside of the CE. Theoretically, if these putative neo-epitopes share homology with *Macaca mulatta* proteins, the elicited responses may result in recognition of self-molecules. A search through the *M. mulatta* proteome indicated that few proteins (Fig. 5) shared limited homology with the junction and linkers sequences, spanning only five to seven AA, and lack the fully homologous length to elicit potentially self-reactive CD4^+^ or CD8^+^ T-cell responses. Similarly, comparison to the human proteome shows a very low extent of homologies (Fig. 5).

Peptides were designed covering the linkers and the adjacent HIV CE sequences, and these peptides were used to analyze T-cell responses by flow cytometry in eight macaques that received two priming vaccinations with HIV CE DNA. It was found that the majority (5/8) of the animals were negative for T-cell responses targeting the linker peptides (Table 6). Three macaques showed positive T-cell responses (linker peptides L4-5, L5-6, and L7-1) recognizing either one (L862 and R315) or two peptides (P307). L862 and P307 scored positive for the peptide covering the linker between CE4 and CE5, and R315 scored positive for the peptide covering the linker between CE5 and CE6, but these responses are likely the result of recognition of the AA sequence overlapping with the adjacent CE5. Only P307 scored positive for the peptide covering the junction between CE1 and CE7, and none of the neighboring CE showed positive responses. This peptide shares only five AA with a predicted *M. mulatta* proteome (Fig. 5). Together, these data suggest that there is no significant recognition of any neo-epitopes created by the linkers, and there is low homology to the human or macaque proteome.

**Table 6.** Frequency of antigen-specific IFN-γ^+^ T cells upon stimulation with the indicated peptides in PBMC from macaques receiving two HIV CE DNA vaccinations

	*Peptides spanning:*												
*Animal*	*CE2*^a^	*L2-3*^b^	*CE3*^a^	*L3-4*^b^	*CE4*^a^	*L4-5*^b^	*CE5*^a^	*L5-6*^b^	*CE6*^a^	*L6-7*^b^	*CE7*^a^	*L7-1*^b^	*CE1*^a^
L862	0.16	—	0.02	—	—	0.15	0.06	—	—	—	—	—	—
M166	0.07	—	—	—	—	—	0.1	—	0.45	—	—	—	—
M695	—	—	0.02	—	—	—	0.12	—	0.06	—	—	—	—
R279	—	—	—	—	0.03	—	0.02	—	0.03	—	—	—	—
R315	—	—	—	—	—	—	0.08	0.05	—	—	0.02	—	—
P302	—	—	0.01	—	—	—	0.1	—	0.01	—	—	—	—
P307	—	—	0.50	—	—	0.25	0.23	—	0.04	—	—	0.1	—
P308	—	—	0.05	—	—	—	0.02	—	—	—	—	—	—

Analysis performed weeks after 2nd vaccination with p24CE DNA ^a^using peptide pool (15-mer overlapping by 11 AA and 10-mer overlapping by 9 AA) covering the indicted CE, and ^b^using a single peptide covering the linker and flanking CE, as described in Fig. 5.

## Discussion

This study monitored the magnitude, cytotoxic potential, and longevity of the CE-specific T-cell responses elicited by Gag CE DNA vaccine regimens in rhesus macaques. All animals were immunized according to a schedule that includes priming with CE DNA followed by booster immunizations with a DNA that always contained full-length Gag, a regimen previously identified to maximize magnitude and breadth of the CE immunity.^23,35^ HIV CE DNA and its homologous SIV CE DNA vaccine induced robust CE-specific T-cell responses, reaching up to around 4% of total circulating T lymphocytes in blood, and these responses were long-lasting, being detected around 2 years after the last vaccination. No adverse effects were observed in any of the vaccinated animals during the complete follow-up period. Presently, the HIV CE DNA prime/CE + *gag* DNA co-delivery vaccine regimen is being tested in a Phase I clinical trial (HVTN 119) in healthy volunteers.

This study expanded the CE DNA vaccine regimen by exploring the inclusion of rMVA62B booster vaccination after a long rest period (around 1.5 years). The concept of DNA prime-rMVA boost has been shown to induce promising immune responses in several HIV clinical trials,^48–58^ and a durability of cellular and humoral responses was reported.^48,50,56^ Given the immunodominant nature of the T-cell responses targeting variable regions, which leads to poor CE-specific responses in many animals vaccinated with full-length Gag immunogens,^17^ a question to be addressed was whether rMVA expressing full-length immunogens would boost CE-specific T-cell responses. It was found that rMVA62B booster vaccination potently increased CE-specific T-cell responses. In two animals, a gain of an additional CE response was noted, as also observed in a prior study with a booster vaccination including *gag* DNA.^17,35^ The possibility cannot be excluded that this reflects an increase in pre-existing CE DNA-induced responses that were below the threshold of the assay.

Importantly, both booster vaccination with CE DNA as well as with rMVA induced high numbers of cytotoxic T cells, characterized by GrzB, perforin secretion, and expression of CD107a as a result of degranulation.^82,83^ Boosting with rMVA62B as well as with p24CE DNA resulted in a significant increase of the CE-specific T cells present at the time of the booster vaccination, but this increase did not broaden the number of CE recognized by each animal. Importantly, no negative effect was observed on CE T-cell recall responses comparing CE DNA versus the rMVA62B booster vaccinations. Thus, expression of additional immunogens (*i.e.*, Pol and especially Env) in rMVA62B had no negative impact on boosting the responses targeting the conserved elements. This part of the study resolved one concern, since negative interference by Env epitopes on Gag T-cell response development has been observed in mice, macaques, and humans.^61–63,84^ These data show that priming with the subdominant CE regions expressed from the optimized plasmid DNA permanently altered the immune hierarchy. It was initially shown that CE DNA primed responses could be greatly increased upon full-length *gag* DNA boost in all the vaccinated macaques, although vaccination with full-length *gag* DNA induced CE responses in only around 50% of the animals, and these responses were low and had a narrow breadth among the CE (median one CE/animal). This finding suggests that epitopes located outside of CE within the variable regions of Gag prevent the development of responses targeting the subdominant highly conserved CE regions. Vaccination regimens including CE DNA prime followed by CE + *gag* DNA co-delivery boost provide a successful regimen that allows the development of highly cytotoxic T-cell responses to subdominant CE epitopes. The addition of rMVA62B booster vaccination represents a beneficial expansion of the CE DNA platform for future clinical trials with application in HIV prevention and therapy.
